# Nanoscopic spine localization of Norbin, an mGluR5 accessory protein

**DOI:** 10.1186/1471-2202-15-45

**Published:** 2014-03-26

**Authors:** Linda Westin, Matthias Reuss, Maria Lindskog, Anita Aperia, Hjalmar Brismar

**Affiliations:** 1 Department of Women’s and Children’s Health, Science for Life Laboratory, Karolinska Institutet, 17165 Solna, Sweden; 2 Department of Applied Physics, Science for Life Laboratory, Royal Institute of Technology, 10691 Stockholm, Sweden; 3 Department of Neuroscience, Karolinska Institutet, 17177 Stockholm, Sweden

**Keywords:** Super-resolution microscopy, Colocalization, Norbin, Dendritic spines

## Abstract

**Background:**

Norbin is a neuron-specific, cytosolic protein that interacts with the metabotropic glutamate receptor 5 (mGluR5) and has a profound impact on mGluR5 signaling. Yet, little is known about its synaptic distribution.

**Results:**

Here we have analyzed the spatial relationship between Norbin, postsynaptic density protein 95 (PSD-95), actin and mGluR5 in spines using super-resolution microscopy. Norbin was found to have a high degree of colocalization with actin and a lower degree of colocalization with PSD-95. Co-immunoprecipitation studies confirmed that interaction occurs between Norbin and actin, but not between Norbin and PSD-95. Norbin was also found to have a high degree of colocalization with the perisynaptically located mGluR5. Findings based on structured illumination microscopy (3D-SIM) of exogenous expressed Norbin-GFP were confirmed by stimulated emission depletion microscopy (STED) of immunolabeled endogenous Norbin.

**Conclusions:**

Norbin associates with actin rather than with PSD-95 in dendritic spines. Results regarding protein localization and colocalization performed with conventional confocal microscopy must be interpreted with great caution. The now available super-resolution microscopy techniques provide more accurate information about sub-cellular protein localization than previously was possible.

## Background

Norbin/Neurochondrin is a cytosolic, neuron-specific, somatodendritic protein abundantly expressed in the central nervous system (CNS) [1]. We have previously reported that Norbin acts as an accessory protein to the metabotropic glutamate receptor 5 (mGluR5). Norbin binds to the membrane proximal region of the C-terminal of mGluR5. The interaction between Norbin and mGluR5 results in increased cell surface expression of mGluR5 and augmentation of mGluR5 mediated signaling. Norbin conditional knockout mice with a forebrain specific deletion exhibit impaired mGluR5 dependent long-term potentiation (LTP) and long-term depression (LTD) in hippocampus and a phenotypic behavior similar to what is found in rodent models of schizophrenia [2].

mGluR5 is accumulated in spines, where it is concentrated in the perisynaptic annulus surrounding the postsynaptic density (PSD) [3,4]. Accessory proteins in spines play an essential role in regulating the availability and confinement of glutamate receptors [5,6]. The physiological effects of the interaction between Norbin and mGluR5 suggest that Norbin plays an important role in the modulation of mGluR5 function in the excitatory synapse. Little is however known about the localization of Norbin inside the spine.

Owing to the diffraction limit of light, the resolution in conventional fluorescence microscopy is limited to ~200 nm. In submicrometer-sized spines, virtually all proteins will therefore appear as more or less colocalized, which prevents a detailed analysis of spatial relationships. This technical hurdle has now to some extent been overcome by the advent of super-resolution (nanoscopy) methods, including structured illumination microscopy (SIM), stimulated emission depletion (STED) and single-molecule localization methods (PALM, STORM, GSDIM etc.) (reviewed in [7]). Here we have studied the localization of Norbin in relation to PSD-95, actin, Homer1C and mGluR5 in spines using 3D-SIM [8] and STED [9]. We have also analyzed the confinement of Norbin in spines, by comparing its mobility to a reference cytosolic protein by fluorescence recovery after photobleaching (FRAP).

## Methods

### Primary hippocampal cultures and transfection

All animal experiments were approved by the Institutional Animal Care and Use Committee of the Karolinska Institutet. Primary hippocampal cultures were prepared from E18 Sprague Dawley embryos of either sex as previously described [10] with the following modification: twice a week, half the media was changed to Neurobasal (Invitrogen) with 2% B27 (Invitrogen), 1% penicillin/streptomycin and 0.5 mM L-glutamine (Sigma-Aldrich). After 20–22 days in culture, cells were transfected using Lipofectamine 2000 (Invitrogen). 24–48 hours after transfection cells were collected for experiments.

Constructs: Norbin-GFP, mGluR5-mCherry and mCherry [2], PSD-95-mCherry and PSD-95-BFP [10,11], Homer-DsRed (kindly provided by Dr. Daniel Choquet) and LifeAct-mCherry [12] (Addgene, plasmid 40908).

### Immunocytochemistry

Cells were rinsed briefly in Krebs Ringer Buffer, consisting of, in mM, 110 NaCl, 4 KCl, 1 NaH_2_PO_4_ · H_2_O, 25 NaHCO_3_, 1.5 CaCl_2_ · H2O, 1.2 MgCl_2_ · 6H2O, 10 D-glucose and 20 Hepes, and fixated with 4% paraformaldehyde (Sigma-Aldrich) in PBS for 10 minutes at room temperature. Cells were rinsed with PBS followed by 2 minutes of permeabilization using 0.2% Triton X-100 (Sigma-Aldrich) in PBS. Blocking was done with 10% normal goat serum (NGS, Jackson ImmunoResearch Laboratory Inc.) for 1 hour, followed by incubation with primary antibodies diluted in PBS with 5% NGS for 1 hour at room temperature. After repeated rinsing, cells were incubated with secondary antibodies in PBS with 5% NGS for 1 hour at room temperature. Cells were rinsed repeatedly and mounted in Prolong Gold antifade reagent (Invitrogen).

### Antibodies

Primary antibodies: anti-Norbin rabbit polyclonal antibody which specificity has been tested in Norbin knockout mice [2] (kind gift from Prof. Paul Greengard), anti-PSD-95 mouse monoclonal, (1:500, Abcam), anti-PSD-95 rabbit polyclonal (1:500, Abcam) and anti-actin mouse monoclonal (1:3000, BD Transduction Laboratories). Secondary antibodies: Abberior STAR 440SX goat anti-mouse IgG (1:50, Abberior), Abberior STAR 512SX goat anti-rabbit IgG (1:50, Abberior), Alexa Fluor 488 goat anti-rabbit (1:500, Invitrogen) and Alexa Fluor 568 goat anti-mouse (1:500, Invitrogen).

### Confocal imaging

Imaging and photobleaching was done on a Leica TCS SP5 CW microscope with a 63x/1.4 NA oil objective (Leica). 488 nm and 561 nm excitation wavelengths were used and detection was done at 495–555 nm and 570–650 nm in LAS AF software (Leica). For simultaneous FRAP measurements 128 × 128 pixel images were acquired of spines or 2.5 μm dendritic segments with a pinhole of 2 airy units as follows (image acquisition interval): 5 frames baseline (0.5 s), 5 frames bleach (0.21 s), 5 frames post-bleach (0.21 s) and 10 frames post-bleach (5 s). Cells were kept in KREBS solution at 37°C. Analysis was done in a custom written Matlab (The MathWorks) script. The mobile pool was calculated as the mean of the last two measured intensity values. The half time of recovery was derived from a linear fit between the two measured intensity values closest to 50% the mobile pool.

For intensity comparisons between spines and dendrites in Norbin-GFP and mCherry co-transfected neurons, z-stacks of 512 × 512 pixel images were acquired, with a step size of 0.38 μm and a pinhole of 1 airy unit. Line regions over spine heads and adjacent dendritic segments were selected in maximum projections of the z-stacks and the mean intensities were calculated using ImageJ (http://rsbweb.nih.gov/ij/).

### 3D structured illumination microscopy (3D-SIM)

Transfected cells were fixated in 4% paraformaldehyde and mounted in Prolong Gold. 3D-SIM imaging was performed using a Plan-apochromat 63X/1.4 NA oil objective on an ELYRA PS.1 (Carl Zeiss) microscope. Excitation wavelengths were 488 nm and 561 nm, with detection at 495–575 nm and 570–650 nm respectively. In the case of triple labeling an additional wavelength of 405 nm was used with detection at 420–480 nm. 1002 × 1004 pixel images, averaging over 4 frames were acquired in 3 rotations. Final images were reconstructed using ZEN 2011 software (Carl Zeiss). The ELYRA PS.1 system was calibrated using fluorescent beads (40 nm), yielding a lateral resolution of <100 nm and an axial resolution of ~275 nm.

### STED

**Figure 1:**
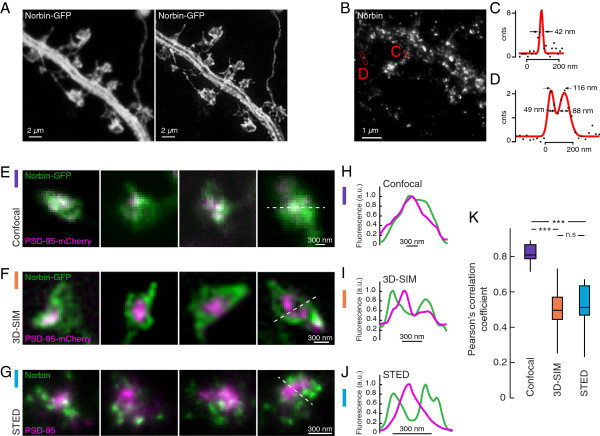
**Super-resolution reveals that Norbin does not colocalize with PSD-95. A)** Confocal (left) and 3D-SIM image of Norbin-GFP distribution in a dendrite with spines. **B)** STED image of immunolabeled Norbin in dendrite and spines. **C)** Line profiles taken from B) (dots) fitted with a Gaussian function (red) indicate a resolution of ~40 nm in the Norbin channel. In **D)** two emitters 116 nm apart have been clearly separated. For the profiles, a patch was taken from the image as indicated in **B)** and integrated along the shorter edge. **E)** Confocal recordings of Norbin-GFP and PSD-95-mCherry show a complete colocalization in spines. **F)** Using 3D-SIM, the distributions of Norbin-GFP and PSD-95-mCherry are resolved. **G)** Immunolabeled endogenous Norbin and PSD-95 recorded using stimulated emission depletion (STED) show the same overall distribution as 3D-SIM images of exogenous expressed Norbin and PSD-95. **H-J)** Intensity profiles along the dashed lines over spine heads in ***A***-***C*** illustrate that confocal microscopy does not have the resolving power of 3D-SIM and STED to identify the discrete localizations of Norbin (green) and PSD-95 (magenta). **K)** Norbin is non-uniformly distributed in spines and has a low degree of colocalization with PSD-95, reflected in a median Pearson’s correlation coefficient (PCC) of 0.49 (n = 27) for 3D-SIM and 0.50 (n = 17) for STED. Compared to the 3D-SIM and STED PCCs, the confocal recordings gave a significantly higher median PCC of 0.81 (n = 15) (***p < 0.00001, Kruskal-Wallis test). The PCCs between the 3D-SIM and STED images are not different (*n.s p* > 0.5, Kruskal-Wallis test). n = number of analyzed spines.

EasySTED [13] was extended to two colors for this work. In brief, excitation light from synchronized 470 and 510 nm pulsed diodes (LDH-P-C-470B/-510B, Picoquant) was combined with depletion light from a 592 nm continuous wave laser (MPBC), coupled via an optical fiber (P1-488 PM-FC-2, Thorlabs) to a beam scanner (YANUS, Till Photonics) into the microscope stand (DMI6000CS, Leica Microsystems) equipped with a 100x/1.4 Oil objective lens (Leica). Underneath the objective lens, a segmented wave plate (Abberior GmbH) selectively re-shaped the 592 nm depletion beam into a bright ring with a central intensity zero. Emission was separated by a 550/49 nm beam splitter (AHF) and guided to an avalanche photo diode (SPCM-AQRH-14-FC, Perkin-Elmer) via a multi-mode fiber (Thorlabs, ~1.5 Airy units). A notch filter for 594 nm (AHF) suppressed residual STED light. Excitation light pulses were alternating between 470 and 510 nm, providing excitation for the two channels in sequence. The signal from the detector was for each excitation pulse separated into two channels by time gated detection in (electronics by MPI for Biophysical Chemistry, Göttingen). To further increase resolution and reduce background, the ~5 ns wide detection windows were delayed ~1 ns with respect to the respective excitation pulses [14]. Resolution was assessed by fitting a Gaussian function to line profiles of spots in the Norbin channel, with ~40 nm full width at half maximum (FWHM) (Figure 1C,D). We used a PCI-6259 board (National Instruments) and the software Imspector (MPI) to drive the scanner and for data acquisition.

### Co-immunoprecipitation and PSD fractions

Co-immunoprecipitation experiments were performed on hippocampi lysate from Sprague Dawley rats as previously described [15] using antibodies for Norbin, actin and PSD-95. PSD fractions were prepared as previously described [16]. Briefly, brain homogenate was fractionated by differential centrifugation and the PSD fraction was obtained through centrifugation in a sucrose gradient. The proteins were separated on a bis-tris gradient gel, transferred to a membrane and immunoblotted against PSD-95 and Norbin.

### Statistical analysis

Non-parametric Wilcoxon rank sum test was used to test the significance of intensity differences between Norbin and mCherry in dendrites and spines. For analyzing colocalization using Pearson’s correlation coefficient (PCC), we used custom software written in Matlab. Individual spines were selected and cropped, normalized and thresholded using Otsu’s method. The PCC for the correlation between the two channels was then computed. FRAP and PCC data was analyzed using non-parametric Kruskal-Wallis test with Bonferroni’s correction for multiple comparisons.

## Results

**Figure 2:**
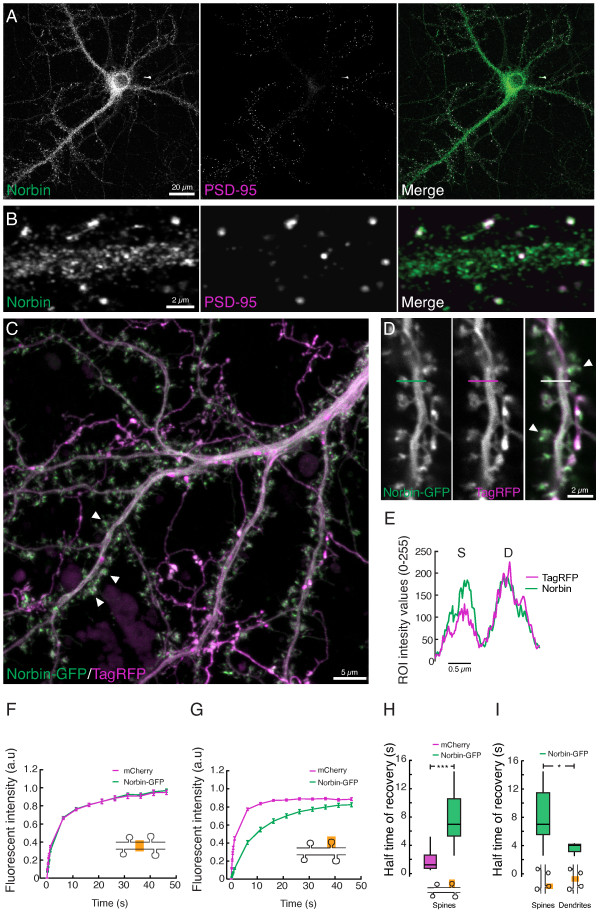
**Norbin localizes to and accumulates in dendritic spines of hippocampal neurons. A)** Confocal images of neurons showing immunolabeling of Norbin and PSD-95. Norbin is localized in the soma and dendrites. **B)** A higher magnification shows that Norbin is localized to dendritic spines. **C-D)** Confocal images of neurons co-transfected with Norbin-GFP and TagRFP showing Norbin accumulated in spines (arrows). **E)** An intensity plot of the line in *D* shows the relative accumulation of Norbin-GFP in spines. **F-I)** Fluorescent recovery after photobleaching (FRAP) experiments in neurons co-transfected with Norbin-GFP and mCherry. **F)** In dendrites, there was no difference in recovery half times between Norbin and mCherry (t_Norbin_ = 3.7 ± 0.2 s, t_mCherry_ = 3.2 ± 0.2 s, mean ± SEM, *p* > 0.5, Kruskal-Wallis test, n = 11). **G, H)** In spines, recovery half times were significantly different between Norbin and mCherry (t_Norbin_ = 8.0 ± 0.4 s and t_mCherry_ = 1.9 ± 0.2 s, mean ± SEM, ***p < 0.0001, Kruskal-Wallis test, n = 59). **I)** Norbin-GFP had a longer half time of recovery in spines than in dendrites (8.0 ± 0.4 s vs. 3.7 ± 0.2 s, mean ± SEM, *p < 0.05, Kruskal-Wallis test).

The endogenous expression pattern of Norbin was identified in hippocampal neurons immunolabeled for Norbin and double-stained for PSD-95, used as a marker for excitatory synapses (Figure 2A,B). Norbin immunofluorescence was observed in the soma, dendrites, spines and axons. The expression pattern of GFP-fused Norbin (Norbin-GFP) in transfected neurons was similar to the expression of endogenous Norbin. To examine whether Norbin accumulates in spines we compared the fluorescent intensity between spines and dendrites for Norbin-GFP and two cytosolic fluorescent proteins, TagRFP and mCherry (Figure 2C-D). An intensity profile along a line over a spine and its adjacent dendrite (Figure 2D) shows that the relative fluorescent intensity between spines (I_S_) and dendrites (I_D_) was larger for Norbin-GFP than for TagRFP and mCherry (Figure 2E). The ratio of the spine to dendrite fluorescent signal was 33% larger for Norbin-GFP than for mCherry ((I_S-Norbin_/I_D-Norbin_)/(I_S-mCherry_/I_D-mCherry_) = 1.33) (n = 307, p < 0.0001, Wilcoxon rank sum test). The difference in the relative fluorescent intensities between Norbin and reference fluorescent proteins suggests that Norbin is accumulated in spines.

To further explore the possibility that Norbin may be confined in spines, we compared the mobility of Norbin-GFP and mCherry in dendrites and spines by performing simultaneous FRAP experiments in Norbin-GFP and mCherry co-transfected neurons (Figure 2F-I). The recovery after photobleaching a 2.5 μm dendritic segment was fast for both Norbin-GFP and mCherry (Figure 2F). The half time of recovery were t_Norbin_ = 3.7 ± 0.2 s and t_mCherry_ = 3.2 ± 0.2 s (mean ± SEM). The mobile pools were highly similar (M_Norbin_ = 0.96 ± 0.02 and M_mCherry_ = 0.95 ± 0.03, mean ± SEM). In spines, the half time of recovery for Norbin-GFP was markedly shorter than for mCherry (t_Norbin_ = 8.0 ± 0.4 s, t_mCherry_ = 1.9 ± 0.2 s, mean ± SEM, p < 0.0001, Kruskal-Wallis test) (Figure 2G,H). Moreover, the half time of recovery for Norbin was significantly different between spines and dendrites (8.0 ± 0.4 s vs. 3.7 ± 0.2 s, mean ± SEM, p < 0.05, Kruskal-Wallis test) (Figure 2I). The difference in half time of recovery between spines and dendrites for mCherry was not significant. The mobile pool of Norbin in spines was smaller than in dendrites M_spines_ = 0.82 ± 0.02 and M_dendrites_ = 0.95 ± 0.02 respectively (mean ± SEM, p < 0.01, Kruskal-Wallis test).

When neurons co-transfected with Norbin-GFP and PSD-95 fused to mCherry (PSD-95-mCherry) were imaged using conventional confocal microscopy, Norbin appeared to have a uniform distribution in the spine head and to colocalize with PSD-95 (Figure 1E,H). We used Pearson’s correlation coefficient (PCC) to quantify the degree of colocalization. A PCC close to one indicates a positive correlation between the protein distributions, and a PCC close to zero indicates that the protein distributions are uncorrelated. The median PCC for Norbin and PSD-95 as measured with confocal microscopy was 0.81 (Figure 1K). Next, we used super-resolution microscopy to study the colocalization between Norbin and PSD-95 (Figure 1F,G). Using 3D-SIM, the distributions of Norbin-GFP and PSD-95-mCherry could be resolved and revealed a non-uniform distribution of Norbin that surrounded, rather than colocalized with, PSD-95 (Figure 1F,I). Consequently the PCC was lower (median PCC = 0.49) (Figure 1K). Considering that exogenous expression may alter protein distribution, we immunolabeled neurons for Norbin and PSD-95 to compare the degree of colocalization between exogenous and endogenous expression. The fluorescence signal from immunolabeled Norbin was, not unexpected, weaker than the signal from Norbin-GFP. SIM is dependent on a good signal-to-noise ratio and could in this situation not substantially improve the resolution. We therefore used STED microscopy for this control experiment. Endogenous Norbin showed a similar low colocalization with PSD-95 as the exogenous expression did (Figure 1G,J). The endogenous expression appeared as more discrete or punctate than the exogenous Norbin-GFP. The overall localization of Norbin and the spatial relation between Norbin and PSD-95 was however highly similar between 3D-SIM and STED generated images, also reflected in closely matching PCCs (Figure 1K).

**Figure 3:**
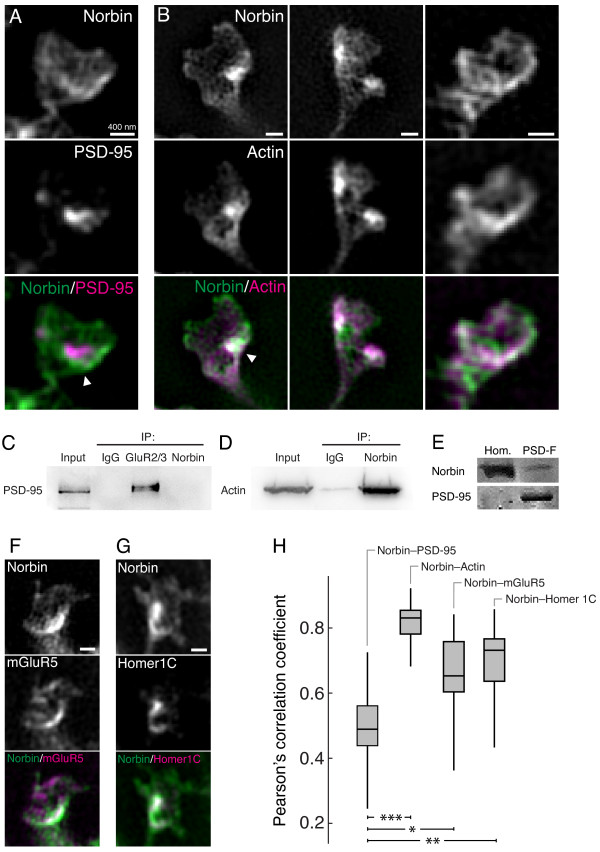
**Spines of neurons co-transfected with Norbin-GFP and either PSD-95-mCherry, LifeAct-mCherry, mGluR5-mCherry or Homer1C-DsRed imaged with 3D-SIM. A)** Norbin clusters close to, but do not colocalize with, PSD-95 (arrow), **B)** Norbin and actin show a high degree of colocalization and cluster at proximal sites, frequently towards the base of the spine head (full arrow). **C)** Norbin and PSD-95 do not immunoprecipitate. **D)** Norbin and actin do immunoprecipitate. **E)** Western blots of brain homogenate (Hom.) and PSD fractions (PSD-F) show that Norbin is absent in the PSD fraction. **F)** Norbin and mGluR5 colocalize in spines. **G)** Norbin and Homer1C colocalize in spines. **H)** Median PCC is significantly lower for Norbin-PSD-95 (PCC = 0.49, n = 27) than for Norbin-Actin (PCC = 0.83, n = 27, ***p < 0.00001), Norbin-Homer1C (PCC = 0.73, n = 25, **p < 0.001) and Norbin-mGluR5 (PCC = 0.65, n = 21, *p < 0.05), Kruskal-Wallis test. Scale bars = 400 nm. n = number of analyzed spines.

As shown in Figure 3A, Norbin clustered at sites close to, but not overlapping with, PSD-95. The PSD and cytoskeletal actin are two of the major structural elements in the spine. To examine how Norbin localizes in relation to actin, neurons were co-transfected with Norbin-GFP and LifeAct-mCherry and imaged using 3D-SIM (Figure 3B). Norbin and actin were highly colocalized in the spine head (median PCC = 0.83) (Figure 3H). The PCC for Norbin and actin was significantly different from the PCC for Norbin and PSD-95 (p < 0.00001, Kruskal-Wallis test) (Figure 3A,H). Norbin and actin clustered at overlapping sites in the spine head (Figure 3B). To further examine the likelihood for an interaction between Norbin and PSD-95, and between Norbin and actin, we performed co-immunoprecipitation experiments. Precipitated Norbin did not interact with PSD-95 (Figure 3C), and precipitated PSD-95 did not interact with Norbin (data not shown), while precipitated Norbin did interact with actin (Figure 3D). As an additional confirmation we prepared PSD fractions using ultracentrifugation. As shown in the Western blot (Figure 3E), PSD-95 was highly enriched in these fractions, whereas Norbin was absent.

Next, we studied how Norbin localized in relation to mGluR5 and to another well-studied mGluR5 adaptor protein, Homer1C. Neurons were co-transfected with Norbin-GFP and with either mGluR5 fused to mCherry (mGluR5-mCherry) or Homer1C fused to DsRed (Homer1C-DsRed) and their localization was studied in spines using 3D-SIM (Figure 3F,G). Norbin and mGluR5 displayed a higher degree of colocalization than Norbin and PSD-95, reflected in a significantly larger median PCC of 0.65 (p < 0.05, Kruskal-Wallis test) (Figure 3F,H). Homer1C and Norbin also showed a high degree of colocalization. The median PCC was 0.73 (Figure 3G,H), significantly larger than the median PCC for Norbin and PSD-95 (p < 0.001, Kruskal-Wallis test).

**Figure 4:**
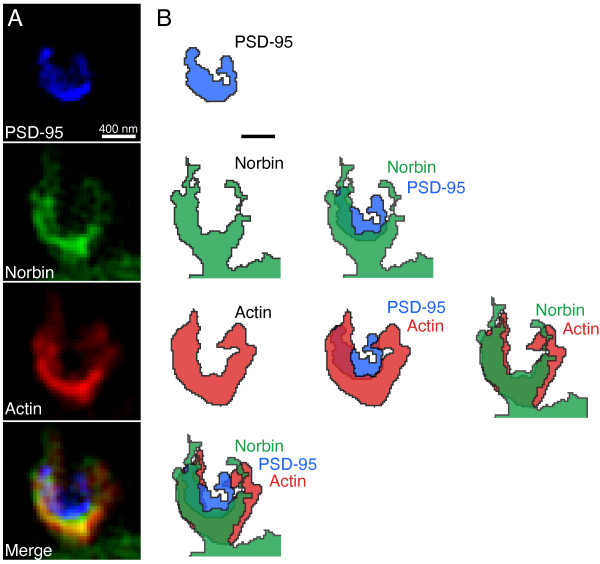
**Norbin overlaps with actin but not PSD-95 in spines. A)** 3D-SIM recording of a spine from a neuron co-transfected with PSD-95-BFP, Norbin-GFP and LifeAct-mCherry. **B)** Segmentation of the PSD-95, Norbin and Actin distributions in ***A*** and their mutual overlap. Scale bar = 400 nm.

To illustrate the differences between the colocalization of Norbin and PSD-95 on one hand and Norbin and actin on the other, we co-transfected neurons with PSD-95 fused to blue fluorescent protein (PSD-95-BFP), Norbin-GFP and LifeAct-mCherry and imaged their localization in spines using 3D-SIM (Figure 4A). While Norbin and actin had a high level of colocalization, both of them only partially overlapped with PSD-95 (Figure 4B).

## Discussion

mGluR5 is anchored in spines and plays a major role for the excitatory synapse via regulation of local protein synthesis and long-term modifications of synaptic strength [3,17-19]. Norbin prolongs the mGluR5 signal and potentiates its long-term effects [2]. Here we show that Norbin can be confined in spines, where it colocalizes with actin rather than with PSD-95. We show that Norbin is perisynaptically located and has a high degree of colocalization with mGluR5. These findings underline the importance of Norbin as an mGluR5 accessory protein in the excitatory synapse.

The organization of proteins in spines is an important determinant of excitatory synaptic function. Much of the information about protein-protein interaction is derived from biochemical studies. To verify this interaction there is a need to study the intact cell. Conventional confocal microscopy is however not always powerful enough for studies of protein interaction and colocalization, as we demonstrate in the current study. Surprisingly, it is rarely realized that due to the diffraction of light, the resolution of a conventional microscope is limited to around ~200 nm. Proteins that are less than 200 nm apart can therefore not be separated, and most proteins will in fact appear to colocalize in the spine head, where the diameter is around 500 nm.

Super-resolution microscopy has emerged as a valuable tool to study protein localization in subcellular compartments of intact cells. In the current study we used two dual color super-resolution approaches. 3D-SIM provides a lateral resolution of ~100 nm and an axial resolution of ~275 nm. STED provides a lateral resolution of ~40 nm, but in current implementations no improvement in the axial direction compared to a confocal microscope.

In a control experiment we compared endogenous and exogenous expression of Norbin using SIM and STED. There were some differences in the distribution of Norbin detected with the two methods. The endogenous distribution of Norbin imaged with STED appeared non-uniform and punctate, while in the 3D-SIM studies the exogenous expression of Norbin appeared more continuous. These differences in appearance might be attributed both to differences in resolution of the microscopy techniques and to differences in labeling methods. 3D-SIM has a lower resolution than STED and gives therefore a slightly blurred image compared to STED. In the 3D-SIM studies, the exogenous expression of Norbin could potentially result in a more homogenous distribution. In the STED study, the antibodies may due to steric hindrance not reach all their target epitopes, which could result in an incomplete labeling. The process of permeabilization during antibody labeling in combination with a mild paraformaldehyde crosslinking of proteins may also cause extraction of proteins and contribute to an underestimation of the total pool of molecules in intact neurons. Despite these technical differences, the results from the 3D-SIM and STED studies were found to correspond well.

The SIM study relied on the expression of a GFP-tagged Norbin. The relevance of localization studies of transfected proteins must always be questioned. The high compliance between the SIM and the STED study, where endogenous Norbin was detected with immunostaining, does however strongly indicate that in this study exogenously expressed Norbin yields a representative view of its endogenous localization and its relationship to PSD-95 and actin.

EM studies have demonstrated a laminar organization of the PSD and that PSD-95 in close proximity to the postsynaptic membrane (~12 nm) [20]. Actin forms long and short-branched filaments that extend from the base of the spine and supports the PSD. Actin is considered to provide activity dependent structural plasticity of the spine and is important for the confinement of perisynaptically located proteins [21,22]. mGluR5 is concentrated in the perisynaptic annulus and at greater distance from the site of glutamate release than the ionotropic glutamate NMDA and AMPA receptors, which are stabilized by PSD-95 [3,4]. Our nanoscopic analysis indicates that Norbin is to a large extent colocalized with both mGluR5 and Homer1C in the perisynaptic region. The mGluR5 adaptor protein Homer is located beneath the superficial layers of the PSD, adjacent to the cytoplasmic border (~60 nm from the active zone) [23]. The relative roles of Norbin and Homer proteins as modulators of mGluR activity, remains to be determined.

## Conclusions

In conclusion, this study has shown that Norbin associates with actin rather than with PSD-95 in dendritic spines. The findings also illustrate that results regarding protein localization and colocalization performed with conventional confocal microscopy must be interpreted with great caution. The now available super-resolution microscopy techniques provide more accurate information about sub-cellular protein localization than previously was possible.

## Competing interests

MR has filed patent applications related to easy- and gated-STED.

## Authors’ contributions

LW participated in design of the study, did the cell culture, co-immunoprecipitation and labeling, carried out imaging and data analysis and drafted the manuscript. MR constructed the easy-STED microscope, carried out imaging and data analysis and helped to draft the manuscript. ML did PSD fractions and analysis. AA and HB conceived the study, participated in its design and coordination and drafted the manuscript. All authors read and approved the final manuscript.
